# On the temporal dynamics of spatial stimulus-response transfer between spatial incompatibility and Simon tasks

**DOI:** 10.3389/fnins.2014.00243

**Published:** 2014-08-19

**Authors:** Jason Ivanoff, Ryan Blagdon, Stefanie Feener, Melanie McNeil, Paul H. Muir

**Affiliations:** ^1^Department of Psychology, Saint Mary's UniversityHalifax, NS, Canada; ^2^Department of Mathematics and Computing Science, Saint Mary's UniversityHalifax, NS, Canada

**Keywords:** speed-accuracy trade-off, stimulus-response compatibility, Simon effect, spatial compatibility, S-R associations

## Abstract

The *Simon effect* refers to the performance (response time and accuracy) advantage for responses that spatially correspond to the task-irrelevant location of a stimulus. It has been attributed to a natural tendency to respond toward the source of stimulation. When location is task-relevant, however, and responses are intentionally directed away (incompatible) or toward (compatible) the source of the stimulation, there is also an advantage for spatially compatible responses over spatially incompatible responses. Interestingly, a number of studies have demonstrated a reversed, or reduced, Simon effect following practice with a spatial incompatibility task. One interpretation of this finding is that practicing a spatial incompatibility task disables the natural tendency to respond toward stimuli. Here, the temporal dynamics of this stimulus-response (S-R) transfer were explored with speed-accuracy trade-offs (SATs). All experiments used the mixed-task paradigm in which Simon and spatial compatibility/incompatibility tasks were interleaved across blocks of trials. In general, bidirectional S-R transfer was observed: while the spatial incompatibility task had an influence on the Simon effect, the task-relevant S-R mapping of the Simon task also had a small impact on congruency effects within the spatial compatibility and incompatibility tasks. These effects were generally greater when the task contexts were similar. Moreover, the SAT analysis of performance in the Simon task demonstrated that the tendency to respond to the location of the stimulus was not eliminated because of the spatial incompatibility task. Rather, S-R transfer from the spatial incompatibility task appeared to partially mask the natural tendency to respond to the source of stimulation with a conflicting inclination to respond away from it. These findings support the use of SAT methodology to quantitatively describe rapid response tendencies.

## Introduction

The spatial configuration of stimuli and responses greatly affects human performance (Fitts and Seeger, 1953; Fitts and Deininger, 1954). Studies of stimulus-response (S-R) compatibility provide an opportunity to explore which sorts of S-R associations are more natural, and perhaps more automatic, than others. *Spatial incompatibility* tasks, where the stimulus location is *task-relevant* and the goal is to respond away from a stimulus, are generally performed more slowly and with greater errors than *spatial compatibility* tasks, where responses are directed toward stimuli (Fitts and Deininger, 1954). Fitts and Deininger proposed that the number of transformations between stimulus and response was a partial determinant of speeded responding under S-R compatible/incompatible conditions. Others have taken a slightly different approach, suggesting that the number or complexity of rules in an incompatibility task is greater than it is in a compatible condition (Duncan, 1977, 1978). It is generally thought that it is easier to respond when there is some kind of conceptual match between stimulus and response features (Kornblum et al., 1990).

The location of a stimulus, even when task-irrelevant, affects spatial responding (Simon and Rudell, 1967; Simon, 1969), suggesting there is some sort of well-established or automatic pathway extending from neural regions responsible for processing stimulus location to neural regions responsible for response selection. The *Simon effect* refers to the performance advantage for spatially corresponding responses over non-corresponding responses, when the location of the stimulus is task-irrelevant. It was originally attributed to “a ‘natural’ tendency to react toward the source of stimulation” (Simon, 1969, p. 175). Dual-route models (de Jong et al., 1994) usually incorporate this natural tendency as a feature of the automatic, or direct, pathway that speeds (corresponding), or slows (non-corresponding), responding. Although other accounts of the Simon effect have emphasized various mechanisms (e.g., see Lu and Proctor, 1995; Proctor, 2011; Van der Lubbe and Abrahamse, 2011; Hommel, 2011 for reviews), most accounts do tend to incorporate some kind of “natural tendency” for location information to influence response selection.

### Transfer of S-R pathway activity across simon and spatial incompatibility tasks

In recent years, there has been growing interest in the transfer of S-R mappings between spatial incompatibility and Simon tasks. Proctor and Lu (1999) demonstrated that the Simon effect reversed (i.e., spatially non-corresponding responses were faster than spatially corresponding responses) when the Simon task was preceded by a spatial incompatibility task. In other studies, transfer from the spatial incompatibility task to the Simon task has eliminated, but not reversed, the Simon effect (Tagliabue et al., 2000). Tagliabue et al. (2000) attributed this discrepancy to the greater number of practice trials in the spatial incompatibility task in Proctor and Lu's (1999) study (~1800 trials) compared to that of their study (72 trials). The reverse (or absent) Simon effect following a spatial incompatibility task has been explained in one of two ways.

The first account of the reverse (or absent) Simon effect following a spatial incompatibility task is, perhaps, the most pragmatic of the two proposals. The Simon effect has routinely been attributed to “automatic” response priming from the corresponding stimulus location. This priming is thought to occur along the direct, spatial S-R pathway. Proctor and Lu (1999) suggested that activation of the direct pathway is not necessarily immutable. In their description of the connectivity between spatial features of the stimulus and the response they state, “[t]hese associations have been described as *unconditional* (de Jong et al., 1994), *permanent* (Barber and O'Leary, 1997), and as being either *hard-wired* or *learned from a lifetime's experience* (Umiltà and Zorzi, 1997). The implication of such descriptions - that the associations are essentially unmodifiable—is incorrect” (Proctor and Lu, 1999, p. 76). Thus, the learned associations from the spatial incompatibility task may simply “overwrite” the direct pathway thereby reversing, or eliminating, the Simon effect.

**Figure 1:**
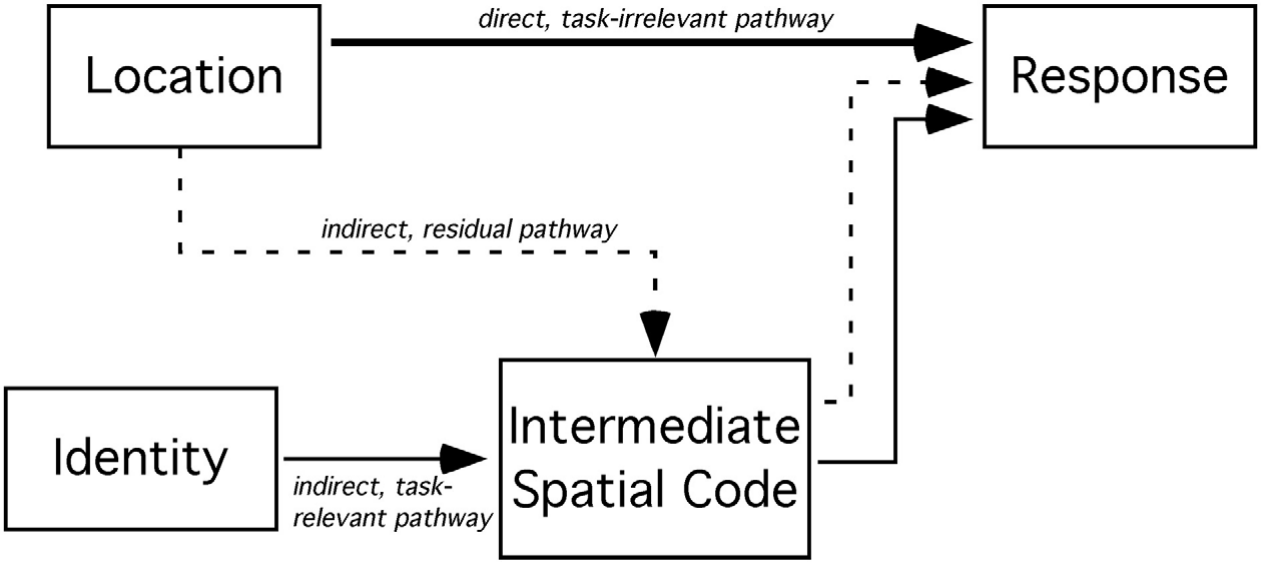
**An illustration of the three S-R pathways in a Simon task modified by a spatial incompatibility task**. The direct, task-irrelevant pathway offers fast connectivity between stimulus location codes and response codes. The indirect, task-relevant pathway between the (non-spatial) identity of the stimulus and the response codes passes through an intermediate translation stage. Lastly, in the case of prior (or co-existent) experience with a spatial incompatibility task, the residual pathway from the location of the stimulus to response codes also passes through an intermediary stage where spatial codes are re-assigned new mappings.

Tagliabue et al. proposed a different account of the effect of a spatial incompatibility task on the Simon effect. Their account includes three pathways (see Figure 1 for a graphical representation of the three pathways). The direct spatial S-R pathway, connecting location stimulus codes directly to response codes, has a quick, yet evanescent, onset. One of the slow, indirect S-R pathways (sometimes called the *conditional route* or the *controlled pathway*) is task-relevant: it translates non-spatial, symbolic stimulus codes to intermediary spatial codes that, in turn, connect to response codes. Dual pathway models have long been presumed to encompass the cognitive architecture necessary for the Simon effect (e.g., de Jong et al., 1994). The other indirect pathway is spatial and is the result of residual activity from the spatial incompatibility task. It is likely slower than the direct spatial pathway. It connects stimulus location information to intermediary spatial codes that, in turn, recode spatial stimulus information for spatial response selection (e.g., left → right and right → left). Tagliabue et al. (2000) argued that this particular model accounts for the time course of the Simon effect, following the performance of a spatial incompatibility task, quite well.

In contrast to the S-R transfer evident from a spatial incompatibility task to a Simon task, there is currently little evidence for S-R transfer from a spatial compatibility task to a Simon task. Proctor and Lu (1999) observed a 21 ms Simon effect following practice in a task with central (neutral) stimuli and a 21 ms Simon effect following practice with a spatial compatibility task. Tagliabue et al. (2000) noted a baseline Simon effect of 38.5 ms (Experiment 6), and a Simon effect of 26.5, 35, and 33 ms (Experiments 3-5) when preceded by spatial compatibility task. Tagliabue et al. (2000) argue that the spatial S-R mappings from a spatial compatibility task cannot further strengthen the direct pathway. Accordingly, the (absent) effect of a spatial compatibility task on the Simon effect provides reasonable experimental control to evaluate the adverse effect of a spatial incompatibility task on the Simon effect.

Interestingly, S-R transfer does not seem to be particular to a set of stimuli as it occurs when different stimuli sets are used across tasks (Proctor and Lu, 1999). S-R transfer also occurs across different stimulus modalities (Tagliabue et al., 2002), although perhaps more weakly, given that the spatial incompatibility task did not reverse the Simon effect in this study. With a sufficient number of practice trials, there is even some evidence for S-R transfer when the spatial incompatibility task is presented along a different spatial axis from the Simon task (e.g., the practice stimuli and responses in the spatial incompatibility task are presented along the horizontal axis while the transfer stimuli and responses in the Simon task are presented along the vertical axis), suggesting that in some cases a S-R rule (e.g., a “respond opposite” procedure) may transfer across tasks (Vu, 2007). S-R transfer from a spatial incompatibility task to a Simon task may be relatively persistent. Transfer effects have been observed when the interval between spatial incompatibility and Simon tasks has ranged from 5 min to days (Tagliabue et al., 2000, 2002). S-R transfer across tasks is also evident in so-called mixing tasks, where the spatial incompatibility task alternates, or is interleaved, with a Simon task (Marble and Proctor, 2000; Proctor et al., 2000).

Despite its ubiquity, there is little known about three facets of S-R transfer between spatial incompatibility and Simon tasks. First, current research has emphasized S-R transfer in one direction (i.e., the effect of practicing a spatial incompatibility task on the Simon effect). Very little is known about the impact of non-spatial, task-relevant S-R mappings in a Simon task on spatial incompatibility tasks. The potential existence of bidirectional S-R transfer has implications for our understanding of the limitations of S-R transfer. Secondly, there has been little work on the time course of Simon effects following transfer from a spatial incompatibility task. Speed-accuracy trade-off (SAT) approaches to the time course of the Simon effect do not possess the same disadvantages as other, more common, RT distributional analyses (e.g.,Zhang and Kornblum, 1997). Lastly, context of the task is known to play a critical role in memory transfer (Smith and Vela, 2001), but its place in S-R transfer has not yet been firmly established.

### Bidirectional S-R transfer

Very little research has explored the effect of task-relevant S-R mappings from a Simon task on performance in a spatial compatibility or incompatibility task. One reason for the paucity of attention on bidirectional S-R transfer is paradigmatic. Practice tasks (e.g., Proctor and Lu, 1999; Tagliabue et al., 2000) typically only include two blocks of trials—practice and test—in a fixed order (i.e., spatial compatibility or incompatibility task followed by the Simon task), thus not permitting an evaluation of the effects of S-R mapping in the Simon task on the spatial compatibility and incompatibility tasks. The other reason that bidirectional transfer is typically not explored is methodological. In mixing tasks (Marble and Proctor, 2000; Proctor et al., 2000), a non-spatial feature of the stimulus informs the participant to perform a left, right or spatial compatibility (or incompatibility) task. For example, the color of the stimulus in Marble and Proctor's (2000) task informed the participant to make a particular response (i.e., a red or green stimulus informed participants to make a left or right response), while another color (white) instructed the participant to make a spatially compatible (or incompatible) response. Accordingly, this particular methodology does not permit the researcher to explore the effect of task-relevant (non-spatial) Simon task S-R mappings on performance in the spatial compatibility or incompatibility tasks.

The exception to this lack of attention to bidirectional transfer is Proctor and Lu (1999; Experiment 2). In their task, participants made left or right responses to letters (S or H) presented to the left or right side of the screen. They practiced this Simon task in three sessions before transfer to a spatial compatibility or incompatibility task. Although the authors initially failed to record letter identity in the transfer session, a subsequent study corrected this oversight and they found no effect of letter identity on RTs within spatial compatibility/incompatibility tasks; however, there was an effect of letter identity on error rate (i.e., there were more errors when the response assigned to the letter was incongruent with the location in both compatibility and incompatibility tasks). It is not clear why the congruency effect only influenced error rates. Proctor and Lu did not discuss the implication of their finding in great detail.

There are a few theoretical implications for considering the effect of task-relevant non-spatial mappings from a Simon task on performance in a spatial compatibility or incompatibility task. Firstly, location information generally precedes selection for color or shape (Hillyard and Munte, 1984). If prior S-R mappings between (slow) non-spatial features do not affect (fast) responses to location, then S-R transfer may hinge on temporal precedence. Second, the lack of evidence for S-R transfer from Simon to spatial compatibility/incompatibility tasks might suggest that S-R transfer is closely tied to spatial features of the stimulus. Lastly, it is possible that non-spatial S-R mappings are relatively weak and, consequently, do not transfer once the task is abandoned. In the mixed-task paradigm (Marble and Proctor, 2000), however, task-relevant S-R mappings from the Simon task should not be completely abandoned in the spatial compatibility/incompatibility tasks because they will be needed once again, once the Simon task cue is reintroduced. In the current study, the stimuli on Simon and spatial compatibility/incompatibility trials were identical in a variant of the mixed-task paradigm to provide a fertile opportunity to detect bidirectional S-R transfer. A cue precedes a block of trials informing participants to engage in a particular task (i.e., a Simon or spatial compatibility/incompatibility task). This methodology allows for an examination of the effects of non-spatial S-R mappings from the Simon task on performance in the spatial compatibility and incompatibility tasks.

### Time course of the simon effect following a spatial incompatibility task: vincentizing reaction times and speed-accuracy trade-offs

The time course of the Simon effect has played a critical role in the theoretical development of purported mechanisms behind the effect (Ridderinkhof, 2002). Although a number of chronometric approaches purport to measure the unfolding of mental processes (Meyer et al., 1988), one approach in particular has been widely used in the Simon effect literature. de Jong et al. (1994) were the first to use *vincentized* RTs (Ratcliff, 1979) to study the time course of the Simon effect. According to this approach, RTs are rank ordered, divided into *bins* (quartiles, quintiles, and deciles are most commonly used), and then averaged within a bin for each condition. When the corresponding mean RT for each bin is subtracted from the non-corresponding mean RT, it is referred to as a *delta plot* (Ridderinkhof, 2002). The delta plot of the Simon effect has been interpreted as a direct measure of task-irrelevant spatial response activity. Most studies of the standard Simon effect have demonstrated negative-going slopes with the delta plot approach (Schwarz and Miller, 2012), although there are some exceptions (see Proctor et al., 2011). The interpretation of the decreasing Simon effect has been controversial, with some suggesting a passive decay of task-irrelevant activity along the direct pathway, while others suggest the direct pathway is actively suppressed (see Proctor et al., 2011, for a review of the literature).

The interpretation of delta plots is not without its challenges (Zhang and Kornblum, 1997; Schwarz and Miller, 2012). Zhang and Kornblum (1997) pointed out that the negative-going slopes of delta plots from Simon tasks simply derive from the shapes of corresponding and non-corresponding RT distributions. In particular, smaller variance in the non-corresponding condition, relative to the corresponding condition, gives rise to a negative-going slope (see also Pratte et al., 2010). Keep in mind that this description of the RT distribution does not, however, presuppose a particular mechanism (Schwarz and Miller, 2012). Moreover, delta plots of RTs do not account for error rates.

Error rates are often considered secondary to RT in many tasks, even though they can reveal valuable information about performance. For instance, Hilchey et al. (2011) recently examined the Simon effect using two different measures of response accuracy within the context of an SAT task. A symbol (i.e., ⊗ or a ⊕) was used to instruct participants to make a left (L) or right (R) response. The location of the task-relevant symbol could be to the left, *l*, or right, *r*, of fixation. Hilchey et al. calculated the sensitivity (*d*') to the task-relevant (identity-based: *d*'_*id*_), and task-irrelevant (location-based: *d*'_*loc*_), features of the target. These calculations were possible because of the orthogonal relationship between the identity (⊗ or ⊕) and location (*l* or *r*) of the stimulus. First, ignoring the spatial correspondence between stimulus and response, *d*'_*id*_ was calculated according to the identity of the stimulus (and task instructions):

(1) $${d^{\prime }}_{id}=\frac{z\left[p(L|⊗\text{​})\right]-z\left[p(L|⊕\text{​})\right]}{\sqrt{2}},$$

where *z*[] is the inverse of the standard normal cumulative distribution. The divisor is a standard correction when the signal detection approach is applied to alternative forced choice designs (Macmillan and Creelman, 2005). The probability of responding with a “left” response given the ⊗ stimulus, *p*(L|⊗), is also a *hit* within the framework of signal detection theory. On the other hand, the probability of responding L given the ⊕ stimulus [*p*(L|⊕)] is a *false alarm* error. Hilchey et al. (2011) observed that *d*'_*id*_ increased with time, presumably reflecting evidence accrual along the task-relevant, indirect, non-spatial S-R pathway.

The second way in which Hilchey et al. (2011) assessed sensitivity was according to the location of the stimulus. Sensitivity to the location of the stimulus (*d*'_*loc*_) was calculated with the signal detection framework, this time ignoring the non-spatial stimulus identity. It was calculated according to the following equation:

(2) $${d^{\prime }}_{loc}=\frac{z\left[p\left(L|l\right)\right]-z[p(L|r)]}{\sqrt{2}}.$$

Here, the probability of responding with a left response, to a stimulus presented on the left side of space, *p*(*L*|*l*), is a *hit*. The probability of responding with a left response to a stimulus on the right, *p*(*L*|*r*), is a *false alarm* error. Hilchey et al. (2011) observed that this measure of sensitivity decreased with time. This measure is strongly related to the performance difference between corresponding and non-corresponding trials (see Hilchey et al., 2011). In signal detection theoretic terms, *d*'_*loc*_ most closely captures Simon's (1969) interpretation of the Simon effect: it reflects the sensitivity to the location of the stimulus. In other words, the *d*'_*loc*_ score presumably reflects the combined impact of the direct, and indirect, spatial S-R pathways (illustrated in Figure 1) on response selection. Although this measure approximated an exponential decay function with response lag (time), this kind of function has yet to be quantitatively fitted to data.

#### Speed-accuracy tradeoffs: methodology and functions

Although there are a number of methodological approaches for measuring SAT functions, the response-signal technique is arguably one of the most common (e.g., Schouten and Bekker, 1967; Reed, 1973; McElree and Carrasco, 1999; Carrasco and McElree, 2001). With this procedure, participants are presented with a target stimulus (usually a visual stimulus) and they withhold responding until the onset of a response signal (usually a simple auditory tone). Following the response signal, there is a short (≤300 ms) window in which responses are collected. Responses that precede the window, or follow it, are typically discarded. Varying the interval between the target onset and the response signal controls reaction time. SAT functions allow for a quantitative description of the time course of an effect as an alternative to the delta plot approach (Pachella, 1974; Wickelgren, 1977; Salthouse and Hedden, 2002). SAT functions typically plot *d*' as a function of response lag (i.e., time). Response lag is the sum of the mean reaction time to the response signal (i.e., RTs within the response window) and the stimulus onset asynchrony (SOA) between the target and the response signal. The following is a general equation for the SAT function (Wickelgren, 1977) that has been widely used to describe the trading relation between speed and accuracy:

(3) $${d^{\prime }}_{id}\left(t\right)=\lambda \left[1-e^{-\beta \left(t-\delta \right)}\right],\;for\;t>\delta ,\;else\;0,$$

where *t* is the mean response lag, λ is the asymptotic *d*' value, β is a rate parameter, and δ is the intercept. This SAT function, describing accumulation of evidence to a maximum, is used to fit many different sorts of SAT datasets and seems to fit just as well as other equations (McElree and Dosher, 1989).

Although the exponential SAT function in Equation 3 is quite common, Wickelgren once suggested that “no one knows the correct mathematical form for the speed-accuracy tradeoff function for any cognitive process, so the exponential approach to a limit… should be taken solely as an example” (Wickelgren, 1977, p. 70). One potentially serious challenge to this function is that, in practice, early data points close to the intercept sometimes rise slowly from the baseline, not as abruptly as is assumed in Equation 3. The standard SAT equation does not account for any changes in *d*'_*id*_ between *t* = 0 and δ (one of the parameters to be determined by the fitting process). Thus, Equation 3 is rather unusual as many psychometric functions generally follow an ogive, or an S-shaped, function (Gescheider, 1997) where there is gradual change in the dependent measure (plotted along the y-axis) at the extremes of the independent variable (plotted along the x-axis). Thus, as an alternative approach to the standard SAT function in Equation 3, it seems reasonable to include gradual, rather than abrupt, evidence accrual into the function. Accordingly, a hyperbolic tangent function might capture the slight accumulation of evidence from an assumed *d*' = 0 (at *t* = 0):

(4) $${d^{\prime }}_{id}\left(t\right)=\frac{\lambda }{2}\left[1+\tanh\left(\frac{t-\omega }{\kappa }\;\right)\right],for\;t\geq \;0,$$

where λ is the asymptotic value, ω is a shift parameter (i.e., reflecting the time at which the function reaches 50% of λ) and κ reflects the speed of the transition from the initial region where *d*' = 0 to the final region where *d*' takes on its asymptotic value of λ. Unlike the standard SAT equation, Equation 4 models the entire timecourse, from *t* = 0 to asymptote (λ). This hyperbolic tangent function produces an ogive-shaped curve that permeates much of psychophysics (Gescheider, 1997).

Neither of these functions, however, adequately captures the decreasing sensitivity to location information Hilchey et al. (2011) observed. However, it does appear that *d*'_*loc*_ may fit a simple exponential decay function:

(5) $${d^{\prime }}_{loc}\left(t\right)=\delta e^{\left(-\beta t\right)},\;for\;t\geq \;0,$$

where δ is the peak *d*'_*loc*_ value at *t* = 0 and β is a decay rate parameter. It has yet to be determined how well *d*'_*loc*_ data fit this function.

The goodness of fit of SAT functions is typically assessed using an adjusted R^2^ (Dosher et al., 2004) which includes a penalty for increasing the number of parameters:

(6) $$R_{adj}^{2}=1-\frac{\sum_{i=1}^{n}{\left(d_{i}-\;\hat{d_{i}}\right)}^{2}/\;(n-k)}{\sum_{i=1}^{n}{\left(d_{i}-\;\bar{d}\right)}^{2}/\;(n-1)}\;,$$

where *k* is the number of free parameters, *n* is the number of data values, *d_i_* are the observed *d_i_* values, ${\hat{d}}_{i}$ are the predicted *d_i_* from the model, and *d* is the mean.

Using SAT functions to explore the time course of spatial information processing in a Simon task has a possible benefit over distributional analyses (e.g., vincentizing or delta plots) in that it captures response *decisions* at a given time and is therefore practically immune to the different distributional properties of corresponding and non-corresponding RTs (Zhang and Kornblum, 1997).

The time course of the Simon effect that follows a spatial incompatibility task is unlike what one usually sees with a standard Simon task. In studies that have included a vincentized analysis of RT, the reverse Simon effect, resulting from prior or concurrent experience with a spatial incompatibility task, *increases* with increasing RT (Marble and Proctor, 2000; Proctor and Vu, 2009). This time course seems rather unnatural, as there is no *a priori* theoretical reason to suppose that a reverse Simon effect should not be actively suppressed or naturally decay with time (but see Tagliabue et al., 2000). The use of vincentized RTs as a measure of time course is convenient, but as previously discussed, it is not without its interpretational challenges. Here, we use SAT functions to explore the full temporal dynamics of the reverse Simon effect that follows from mixing a spatial incompatibility task with a Simon task.

### The role of task context on S-R transfer

Surprisingly, there has been little investigation into the effect of environmental context on S-R transfer effects in Simon tasks. Recognition performance is often best when the testing conditions resemble those in training (e.g., Godden and Baddeley, 1975). Context plays an important role in memory (Smith, 1994; Murnane et al., 1999), perhaps because incidental environmental features are usually encoded with task-relevant information, unless intentionally suppressed (Smith and Vela, 2001). One recent study (Milanese et al., 2011) explored the effect of practicing a spatial incompatibility task with a partner on a subsequent *social* Simon task, also performed with a partner. Like the standard version of this paradigm, where only one individual performs the task, the social Simon effect reverses when it follows practice with a spatial incompatibility task (Milanese et al., 2010). Milanese et al. (2011) observed that switching partners between tasks did not eliminate the reverse social Simon effect. Given that the identity of the partner was not integral to the task, it is likely that it would not be a salient feature of the task context. When the partners changed positions (i.e., from the left side to the right side), however, there was no effect of the spatial incompatibility task on the Simon effect. In this task, one's position relative to the partner is a stimulus feature that is critical to performing the task properly. Thus S-R transfer may depend on task-relevant, salient features.

Another paper (Yamaguchi and Proctor, 2009) considered response mode to be an integral part of context. Yamaguchi and Proctor's (2009) participants performed a spatial incompatibility task by responding to stimuli on a keyboard or a joystick. Participants then performed a Simon task (with a keyboard or a joystick), where the color of the stimulus was task-relevant and the location was task-irrelevant. When the response mode was consistent across tasks the reduction of the Simon effect (from the spatial incompatibility task) was generally greater than when the response mode did not match. Thus, response mode may provide a context that modulates S-R transfer.

There are two reasons to expect a contextual modulation of S-R transfer across tasks in the present study. First, the response-signal methodology (used to acquire SAT functions) is quite different from standard RT tasks in which instructions emphasize both the speed and accuracy of performance. The response-signal methodology includes auditory signals and visual feedback that are not present in the standard RT tasks. These components are necessary to control RT in SAT tasks. Second, previous work has demonstrated a switch cost when switching between tasks with different speed-accuracy instructions (Gopher et al., 2000). This switch cost suggests that SAT settings constitute part of a task-set. Thus, it is expected that when spatial compatibility/incompatibility and Simon task contexts are similar (i.e., they are both SAT or standard RT tasks) maximal S-R transfer should occur.

### The present study

The current investigation used a mixing task, where Simon and spatial compatibility (or incompatibility) tasks were signaled with a task cue and alternated predictably every eight trials. Unlike previous experiments using the mixed-task methodology (Marble and Proctor, 2000; Experiment 1), the stimuli in the present study were identical in both the Simon and spatial compatibility/incompatibility tasks. In Experiment 1, both the Simon and spatial compatibility effects were measured in standard RT tasks. In Experiment 4, they were measured in SAT tasks. To date, no study has used SAT methodology to study the temporal dynamics of S-R transfer from spatial incompatibility tasks to Simon tasks. In Experiment 2, the spatial compatibility/incompatibility task was administered with the response-signal methodology, while the Simon task was a standard RT task. In Experiment 3, the reverse was true. Unlike Experiments 1 and 4, the spatial compatibility/incompatibility and Simon task contexts in Experiments 2 and 3 do not match.

## Experiment 1

Participants were provided with a visual cue every eight trials instructing them to perform the Simon task or the spatial compatibility (or incompatibility) task. The instructions of each task equally emphasized the speed and accuracy of responding. One group of participants performed a spatial incompatibility task with the Simon task while another group performed a spatial compatibility task with the Simon task. The stimuli in all tasks are identical. A cue presented at the onset of a block of trials informed participants of the task to perform. The purpose of this experiment was to (1) replicate the reversal of the Simon effect when paired with a spatial incompatibility task, (2) identify the effect of the task-relevant S-R mappings from a Simon task on spatial compatibility and incompatibility tasks, and (3) determine whether transfer occurs in a version of mixed-task design (Marble and Proctor, 2000; Proctor et al., 2000) where the task is predictably cued and stimuli are identical across tasks.

### Methods

#### Participants

Sixteen undergraduate participants from Saint Mary's University took part in the spatial compatibility condition and sixteen took part in the spatial incompatibility condition. All participants were between 18 and 30 years of age. All experiments were approved by the Saint Mary's University Research Ethics Board (REB) in accordance with the Tri-council Policy Statement on Ethical Conduct for Research Involving Humans (Canadian Institutes of Health Research, Natural Sciences and Engineering Research Council of Canada, and Social Sciences and Humanities Research Council of Canada, Tri-Council Policy Statement: Ethical Conduct for Research Involving Humans, December 2010).

#### Apparatus and stimuli

The experiment was conducted on an Apple iMac G3/400 DV computer, powered by a 400MHz Power PC 750 (G3) processor, running OS9. Superlab (ver 1.75; Cedrus, CA) was used to present stimuli. The experiment took place in a quiet room with ambient lighting. Responses were executed by pressing, with index fingers, the “z” and “/” keys on a standard QWERTY Apple keyboard.

The viewing distance was approximately 57 cm. There were three types of cues: (1) “Sym” (symbol task) to signal the Simon task, (2) “Same” (same sided response) to signal a spatially compatible response, and (3) “Opp” (opposite sided response) to signal a spatial incompatibility task. The task cues were 0.75° vertically and 1.5° (“Sym”/”Opp”) or 2.0° (“Same”) wide. Three horizontally arranged square box outlines (1.2° × 1.2°) were used as placeholders for the stimuli. The peripheral placeholders were 5.3° (edge-to-edge) from the central placeholder. The fixation point, a circle with a diameter of 0.8°, was presented in the center placeholder. The target stimuli, ⊗ and ⊕, were presented within a circle of 1.2° in diameter. These targets were placed inside either the left or right placeholder. All images were black on a white background.

#### Procedure and design

Each participant underwent 128 trials, equally split between Simon and spatial compatibility/incompatibility tasks. All stimuli and responses were equally balanced between left and right positions. The starting task was randomly determined. A block of eight trials in a particular task alternated with roughly half the group starting with the Simon task the rest starting with the spatial compatibility/incompatibility task. Each block of eight trials was preceded by the 900 ms presentation of the task cue. Following the task cue, a trial was presented. The sequence of trial events was as follows: blank screen (300 ms), fixation display (450 ms), and target (until response).

Each group took part in two tasks: Simon and spatial compatibility tasks or Simon and spatial incompatibility tasks. No feedback was provided for these tasks, and participants were told to respond as fast and accurately as possible.

***Spatial compatibilitys task.*** Participants were presented with “Same” cue (900 ms) at the beginning of the first trial for every block of spatial compatibility task trials, indicating response to the same-side as stimulus location. Therefore, stimuli presented on the right of the fixation point required “/” key responses and stimuli on the left required “z” key responses.

***Spatial incompatibility task.*** The spatial incompatibility task was the same as the spatial compatibility task with the following exceptions. Participants were presented with “Opp” cue (900 ms) at the beginning of the first trial for every block of the spatial incompatibility task, indicating response to the opposite-side of stimulus location. Therefore, stimuli presented on the right of the fixation point required left (“z”) key response and stimuli presented to the left of fixation required “/” key response.

***Simon task.*** Participants were presented with “Sym” cue (900 ms) at the beginning of the first trial for every location-irrelevant block, indicating they were to respond to the non-spatial identity of the target (i.e., the symbol). Presentation of the ⊗ stimulus indicated a left response while the presentation of the ⊕ stimulus indicated a right response, regardless of the location of the stimulus.

### Results and discussion

RTs in each condition were subject to a recursive procedure eliminating trials with RTs that were less than or greater than 3.5 *SD*s from the mean. This procedure generally eliminated fewer than 5% of all trials across subjects.

#### Simon task

Table 1 presents the mean RTs for the Simon task. A 2 (Simon correspondence: corresponding and non-corresponding) × 2 (group: spatial compatibility and spatial incompatibility) mixed ANOVA revealed the expected interaction between Simon correspondence and group [*F*_(1, 30)_ = 14.35, *MSE* = 599.93, *p* < 0.001]. There was a standard 29 ms Simon effect in the spatial compatibility group and a -37 ms Simon effect in the spatial incompatibility group. This finding replicates a number of papers in the literature demonstrating a reverse Simon effect when it is presented following, or within the context of, a spatial incompatibility task (e.g., Marble and Proctor, 2000; Tagliabue et al., 2000).

**Table 1:** **RTs in the Simon tasks in Experiments 1 and 2**.

**SOA**	**Compatibility group**			**Incompatibility group**		
	**Corr**.	**Non-corr**.	**Simon effect**	**Corr**.	**Non-corr**.	**Simon effect**
**EXP. 1**						
	570	599	29^**^	602	565	−37^**^
**EXP. 2**						
60	491	508	17	491	506	15
120	491	509	18^*^	509	505	−4
240	483	514	31^**^	509	525	16
360	496	517	21	525	525	0
480	520	548	28^*^	552	568	16
960	546	551	5	539	554	15
1440	517	559	42^**^	547	551	4

SOA, stimulus-onset asynchrony; Corr., Corresponding; Non-corr., Non-corresponding.

* Value is significantly different from zero, p < 0.05.

** Value is significantly different from zero, Bonferroni corrected.

The mean sensitivity (*d*'_*loc*_ and *d*'_*id*_) values for the Simon task are presented in Table 2. The *d*'_*loc*_ and the *d*'_*id*_ were compared with an unpaired *t*-test across the incompatible and compatibility groups. Sensitivity to the task-relevant instructions (*d*'_*id*_) was significantly higher in the compatibility group (*d*'_*id*_ = 2.27) than it was in the incompatibility group (*d*'_*id*_ = 1.73), *t*_(30)_= 3.96, *p* < 0.001. Sensitivity to the location of the stimulus also differed significantly [*t*_(30)_ = 9.92, *p* < 0.001] between the two groups (compatible *d*'_*loc*_ = 0.13; incompatible *d*'_*loc*_ = −0.25). Both of these effects were significantly different from *d*'_*loc*_ = 0 (*p*s < 0.001), suggesting that engaging a spatial incompatibility task reverses the tendency to respond toward the source of stimulation.

**Table 2:** **d' scores in the Simon tasks in Experiments 1–4 as a function of SOA in the other (spatial compatibility and incompatibility) task (Experiment 2), and as a function of SOA in the Simon (SAT) tasks (Experiments 3 and 4)**.

**SOA**	**Compatibility group**		**Incompatibility group**	
	***d'_id_***	***d'_loc_***	***d'_id_***	***d'_loc_***
**EXP. 1**				
	2.27^*^	0.12^*^	1.73^*^	−0.25^*^
**EXP. 2**				
60	1.74^*^	0.21^*^	1.81^*^	−0.04
120	1.97^*^	0.11	1.93^*^	0.00
240	2.12^*^	0.83	2.06^*^	−0.07
360	2.20^*^	0.13^*^	2.24^*^	−0.04
480	2.36^*^	0.06^*^	2.30^*^	0.04
960	2.26^*^	0.03	2.27^*^	0.00
1440	2.20^*^	0.11^*^	2.27^*^	−0.03
**EXP. 3 (SAT)**				
60	0.21^*^	1.04^*^	0.13	0.15
120	0.19	0.72^*^	0.50^*^	−0.18
240	0.86^*^	0.39^*^	0.96^*^	−0.16
360	1.71^*^	0.06	1.82^*^	−0.19
480	2.33^*^	0.10	2.29^*^	−0.04
960	2.88^*^	−0.01	2.72^*^	−0.02
1440	2.90^*^	0.03	2.88^*^	0.00
**EXP. 4 (SAT)**				
60	0.02	1.14^*^	0.06	0.22^*^
120	0.02	1.21^*^	0.26^*^	−0.03
240	0.63^*^	0.62^*^	0.65^*^	−0.04
360	1.60^*^	0.41^*^	1.61^*^	−0.14
480	2.17^*^	0.16^*^	1.84^*^	−0.06
960	2.22^*^	0.26^*^	2.67^*^	−0.05
1440	2.43^*^	0.14^*^	2.57^*^	−0.07^*^

SAT, speed-accuracy trade-off; SOA, stimulus-onset asynchrony.

* Value is significantly different from zero, p < 0.05.

** Value is significantly different from zero, Bonferroni corrected.

The Simon effect reversed when the Simon task alternated with a spatial incompatibility task. No such reversal was evident in the control Simon task that alternated with the spatial compatibility task. This finding is consistent with other studies using a different mixed-task design (Marble and Proctor, 2000; Proctor et al., 2000). The spatial incompatibility task also affected the *d*' measures in the Simon task. Consistent with the pattern of RTs, sensitivity to the location of the stimulus (*d*'_*loc*_) was *positive* in the compatibility group, indicating a tendency to respond *toward* the location of the stimulus. In contrast, the same measure was *negative* in the incompatibility group, indicative of a tendency to respond *away* from the stimulus.

#### Spatial compatibility and incompatibility tasks

Trials were sorted into *congruent* and *incongruent* conditions for each task, where congruency reflects the match between the *response assigned to the identity* of the stimulus (i.e., ⊗ or ⊕) in the Simon task and the *location of the stimulus* in the spatial compatibility (or incompatibilty) task. The RTs were entered into a 2 (congruency: congruent and incongruent) × 2 (group: compatible and incompatible) mixed ANOVA. Only the interaction between congruency and group was significant, *F*_(1, 30)_ = 12.15, *MSE* = 204.97, *p* < 0.005. The congruency effect was *negative* (incongruent − congruent = −14.3 ms) and significant in the compatibility group [*t*_(15)_ = 2.55, *p* < 0.05]. In the incompatible condition, the congruency effect was *positive* (+11 ms), *t*_(15)_= −2.39, *p* < 0.05. The mean RTs are presented in Table 3.

**Table 3:** **RTs in the spatial compatibility and incompatibility tasks in Experiments 1 and 3 as a function of congruency and SOA (Experiment 3) in the Simon task**.

**SOA**	**Compatibility task**			**Incompatibility task**		
	**Con**.	**Incon**.	**Con. Effect**	**Con**.	**Incon**.	**Con. Effect**
**EXP. 1**						
	370	356	−14^**^	381	392	11^*^
**EXP. 2**						
60	278	282	4	319	313	−6
120	290	282	−8	331	332	1
240	284	287	3	331	330	−1
360	290	286	−4	344	343	−1
480	296	293	−3	362	357	−5
960	308	322	14	368	361	−7
1440	291	296	5	348	352	4

Con., congruent; Incon., incongruent; Con. Effect, congruency effect; SOA, stimulus-onset asynchrony.

* Value is significantly different from zero, p < 0.05.

** Value is significantly different from zero, Bonferroni corrected.

As in the Simon task, we compared the measures of identity and location sensitivity (*d*'_*id*_ and the *d*'_*loc*_, respectively) across the incompatible and compatibility groups. There was no difference in *d*'_*id*_ in the compatible (*d*'_*id*_ = 0.13) and incompatibility groups (*d*'_*id*_ = 0.09); however both of these effects were significantly different from 0 (*p* < 0.005), suggesting a small, but significant, sensitivity to the target's identity (an irrelevant feature within the context of the spatial compatibility task). As expected, *d*'_*loc*_ was significantly different [*t*_(30)_ = 27.42, *p* < 0.001] between the compatible (*d*'_*loc*_ = 2.42) and incompatibility groups (*d*'_*loc*_ = −2.56), demonstrating that participants were following instructions. The mean *d*' values are provided in Table 4.

**Table 4:** ***d*' Scores in the spatial compatibility and incompatibility tasks in Experiment 1, as a function of SOA in the Simon (SAT) task in Experiment 3, and as a function of SOA in the spatial compatibility and incompatibility tasks (SAT) in Experiments 2 and 4**.

**SOA**	**Compatibility task**		**Incompatibility task**	
	***d'_id_***	***d'_loc_***	***d'_id_***	***d'_loc_***
**EXP. 1**				
	0.13^**^	2.42^**^	0.09^**^	−2.56^**^
**EXP. 2 (SAT)**				
60	0.00	2.48^**^	0.03	−1.35^**^
120	−0.03	2.70^**^	0.01	−2.10^**^
240	−0.02	2.83^**^	0.02	−2.44^**^
360	0.10^**^	2.71^**^	0.04	−2.75^**^
480	0.07	2.64^**^	0.09	−2.49^**^
960	0.03	2.84^**^	0.03	−2.87^**^
1440	−0.01	2.91^**^	−0.02	−2.84^**^
**EXP. 3**				
60	0.00	2.55^**^	0.01	−2.36^**^
120	0.01	2.65^**^	−0.01	−2.46^**^
240	−0.02	2.71^**^	0.02	−2.66^**^
360	0.04	2.79^**^	0.01	−2.67^**^
480	0.01	2.71^**^	−0.01	−2.77^**^
960	0.01	2.78^**^	−0.01	−2.75^**^
1440	0.01	2.78^**^	−0.03	−2.75^**^
**EXP. 4 (SAT)**				
60	0.02	2.46^**^	0.07	−0.92^**^
120	−0.03	2.81^**^	−0.02	−1.86^**^
240	0.00	2.76^**^	0.03	−2.36^**^
360	0.06	2.38^**^	0.12*	−2.36^**^
480	0.02	2.68^**^	0.06	−2.02^**^
960	0.10	2.46^**^	0.03	−2.72^**^
1440	0.19^*^	2.45^**^	0.14^*^	−2.58^**^

SAT, speed-accuracy tradeoff task; SOA, stimulus-onset asynchrony.

* Value is significantly different from zero, p < 0.05.

** Value is significantly different from zero, Bonferroni corrected.

Previous studies have addressed the effect of spatial compatibility tasks on the Simon effect. However, few investigations have addressed the effect of task-relevant S-R mappings from the Simon task on performance in spatial compatibility or incompatibility tasks. Proctor and Lu (1999; Experiment 2), assessed the effects of repeated practice with a Simon task on performance of a spatial compatibility and incompatibility tasks. While there was no effect of congruency on RTs, they did observe an effect on error rates. In the present study, the congruency effect in the incompatibility group was consistent in RTs and *d*'_*id*_, demonstrating a performance *advantage* when the identity-response mapping in the Simon effect converges with the location-response mapping in the incompatibility task. Interestingly, a different pattern emerged with the compatibility task. While responses were more accurate when the identity-response mapping in the Simon effect was congruent with the location-response mapping in the compatibility task, responses were faster when the mappings were incongruent. This trade-off between accuracy and speed suggests that S-R mappings in a Simon task have distinct effects on performance in spatial compatibility and incompatibility tasks.

## Experiment 2

In Experiment 2, the spatial compatibility and incompatibility tasks were subjected to the response-signal methodology for a SAT analysis. The Simon task was the same as it was in Experiment 1 (i.e., standard RT task) and alternated with the spatial compatibility/incompatibility block across all response-signal SOAs. Thus, in Experiment 2, the Simon and spatial compatibility/incompatibility task contexts did not match.

### Methods

#### Participants

Fifteen undergraduate participants took part in each of the compatible/Simon and incompatible/Simon tasks for course credit and monetary bonuses. To encourage participants to make timely responses in the SAT task, they received a penny for every response that fell within the 240 ms response window and an additional penny for a correct response.

#### Procedure

The general procedure was the same as it was in Experiment 1 with the following exceptions. The Simon task was exactly like it was in Experiment 1, however it alternated with the spatial compatibility or incompatibility task in each block of trials. There were seven blocks of trials with 128 trials each. The response-signal methodology was applied to the compatibility and incompatibility tasks, but not the Simon task. In the spatial compatibility and incompatibility tasks, the ⊗ or ⊕ appeared in one of the two peripheral placeholders, left or right of fixation, for 60ms. As before, the ⊗ and ⊕ stimuli, presented to the left or right, were presented with equal frequencies. The response signal, an auditory tone (44.1 KHz, 15 ms), was presented following the onset of the stimulus after a delay (i.e., the SOA). The SOA was fixed within a block. There were seven SOAs: 60, 120, 240, 360, 480, 960, and 1440 ms. Participants were required to respond within a 240 ms response window following the tone. They were also provided visual feedback with respect to the timing, but not the accuracy, of their response. Participants were presented feedback “HIT” when responding within 240 ms of tone, “MISS” when responding more than 240 ms after the tone, and “TOO SOON” when responding prior to the tone. Thus, responding within the response window took precedence over response accuracy. This prioritization reliably encouraged participants to trade accuracy for speed at the shorter SOAs.

### Results and discussion

The RTs in the Simon task were trimmed as before. The *d*' scores were calculated as they were in Experiment 1. The analysis of the spatial compatibility and incompatibility task was much like other SAT analyses. First, only responses that fell within the 240 ms time window following the tone were analyzed. Second, *response lag* was measured in the SAT task, not RT. Response lag is an estimate of the average response time, relative to the response signal (the tone), within the response window *plus* the SOA (for example, if a response was made 129 ms following the tone when the SOA was 360 ms, the response lag would be 489 ms for that particular trial). Lastly, *d*'_*loc*_ and *d*'_*id*_ were estimated for each SOA. The *d*' estimate for each participant was the mean of a bootstrapping procedure. Ten thousand samples (with replacement) were taken from each SOA using the base number of trials found in the SOA with the most trials discarded (i.e., because the responses fell outside of the response window). This bootstrapping procedure was used to ensure that *d*' values were not artificially deflated across SOAs due to missing trials (i.e., when responses did not fall within the response window). Trials with perfect scores were adjusted according to the conventional 0.5*f* recommendation (Kadlec, 1999).

#### Simon task

RTs are presented in Table 1 as a function of SOA in the spatial compatibility task. The Simon trials were separated according to the spatial compatibility/incompatibility group, correspondence (corresponding and non-corresponding), and the SOA from the spatial compatibility/incompatibility task (60, 120, 240, 360, 480, 960, and 1440 ms) and entered into a 2 × 2 × 7 mixed ANOVA. There was a main effect of correspondence [*F*_(1, 28)_ = 12.32, *MSE* = 2238.14, *p* < 0.005], with a 15ms Simon effect overall. There was also a main effect of SOA [*F*_(6, 168)_ = 8.58, *MSE* = 3294.13, *p* < 0.001], where the overall RT in the Simon task increased with the SOA in the spatial compatibility/incompatibility task. Surprisingly, there was no significant interaction between correspondence and group, although there was a numerical reduction in the Simon effect in the incompatibility group (incompatibility group: 9 ms [*t*_(14)_ = 1.19, *p* > 0.25]; compatibility group: 23 ms [*t*_(14)_ = 4.39, *p* < 0.001]).

The *d*' values are presented in Table 2 as a function of SOA in the spatial compatibility task. The *d*'_*id*_ values were entered into a 2 (group) × 7 (SOA) ANOVA. Only the main effect of SOA was significant, *F*_(6, 168)_ = 8.69, *MSE* = 0.14, *p* < 0.001. With increasing SOA, the *d*'_*id*_ values also increased. This suggests that the speed-accuracy setting in the spatial compatibility/incompatibility task transferred to the Simon task. The *d*'_*loc*_ values were also entered into the same 2 × 7 ANOVA. The main effect of group was significant [*F*_(1, 28)_ = 14.82, *p* < 0.005], demonstrating an overall greater effect of location in the Simon task within the compatibility group (*d*'_*loc*_ = 0.15) than with the incompatibility group (*d*'_*loc*_ = −0.03). The interaction between SOA and group was significant, *F*_(6, 168)_ = 3.08, *MSE* = 0.17, *p* < 0.01. We compared the *d*'_*loc*_ values between compatible and incompatibility groups at each SOA. *d*'_*loc*_ was greater for the compatibility group than the incompatibility group at 60ms [*t*_(28)_ = 3.56, *p* < 0.005], 240 ms [*t*_(28)_ = 2.54, *p* < 0.05], 360 ms [*t*_(28)_ = 4.17, *p* < 0.0005], and 1440 ms [*t*_(28)_ = 3.16, *p* < 0.005].

Unlike Experiment 1, there was no evidence of a reversal of the Simon effect in Experiment 2. In fact, there was surprisingly weak evidence of an influence of the spatial incompatibility task on the Simon effect. Individual mean corresponding and non-corresponding RTs from the Simon tasks for the spatial incompatibility groups in Experiment 1 and 2 were entered into 2 (correspondence) × 2 (Experiment) ANOVA. The interaction between correspondence and Experiment was significant [*F*_(1, 29)_ = 15.82, *MSE* = 501.62, *p* < 0.0005], backing the claim that S-R transfer was not as strong in Experiment 2 as it was in Experiment 1. Moreover, the same analysis on the Simon effect for the spatial compatibility groups revealed no interaction between Experiment and correspondence [*F*_(1, 29)_ = 0.54, *MSE* = 423.05, *p* = 0.47]. This finding supports the idea that there is no S-R transfer from spatial compatibility tasks to Simon tasks (Tagliabue et al., 2000). Together, this supplementary analysis suggests that the context of the task has an important modulating influence on S-R transfer effects from spatial incompatibility tasks to Simon tasks.

#### Spatial compatibility and incompatibility tasks

The performance in the spatial compatibility task proved quite easy, as *d*'_*loc*_ was near ceiling across all SOAs (see Table 4). This suggests that information processing along the direct spatial pathway is very quick. For those in the incompatibility task, *d*'_*loc*_ was slightly impaired at the earliest lags, but still not enough for proper curve-fitting as values were still quite far from chance. We analyzed the spatial compatibility task by entering the *d*' values into a 2 (group) × 7 (SOA) ANOVA. The analysis of *d*'_*loc*_ revealed a main effect of SOA [*F*_(6, 168)_ = 10.33, *MSE* = 0.13, *p* < 0.001], an expected large main effect of group [*F*_(1, 28)_ = 2671.44, *MSE* = 0.52, *p* < 0.001], and the SOA x group interaction [*F*_(6, 168)_ = 25.80, *MSE* = 0.13, *p* < 0.001]. Although the SOA effect was significant in both groups, the difference between *d*'_*loc*_ at the longest SOA and the shortest SOA was much greater in the incompatible condition than it was in the compatible condition (Table 4).

The analysis of *d*'_*id*_ also included SOA and group as factors. Only the main effect of SOA was significant [*F*_(6, 168)_ = 2.30, *MSE* = 0.02, *p* < 0.05]. As seen in Table 4, there was a significant increase in *d*'_*id*_ values at 360 ms, but only in the compatibility group did the *d*'_*id*_ values deviate significantly from zero.

There was little evidence of S-R transfer from the Simon task to the spatial compatibility or incompatibility tasks. The only effect of S-R mappings from the Simon task on the spatial compatibility task (i.e., the congruency effect) occurred at the 360 ms SOA. However, there was no *a priori* reason to expect the effect to be restricted to a single SOA. There was also little reason to expect that S-R transfer would not occur from the Simon task to the spatial incompatibility task. Thus, Experiment 2 did not replicate the observation in Experiment 1 of S-R identity transfer from the Simon task to the spatial compatibility and incompatibility tasks. There are two reasons for this apparent discrepancy. First, the response-signal methodology was applied to the spatial compatibility and incompatibility tasks in Experiment 2, while in Experiment 1 they were standard RT tasks. It is possible, though unlikely, that the effects of S-R transfer are not measurable in SAT tasks. Second, the difference in the context of the task may have hampered S-R transfer. Task contexts were reversed in Experiment 3 to assess this latter possibility.

## Experiment 3

In this experiment the Simon task was an SAT task while the spatial compatibility and incompatibility tasks were standard RT tasks. The SAT functions from the Simon task were analyzed in three ways. First, the *d*'_*id*_ values were analyzed using a hierarchical modeling approach (e.g., see McElree and Carrasco, 1999; Carrasco and McElree, 2001). Second, fits with the standard SAT equation (Equation 3) were compared to the fits achieved with the proposed hyperbolic tangent equation (Equation 4). Lastly, the *d*'_*loc*_ data were fit with an exponential decay function (Equation 5).

### Methods

#### Participants

Fifteen undergraduates took part in each condition (compatible and incompatible) for course credit and monetary incentives (for the SAT task) as in Experiment 2.

#### Procedure

The general procedure was the same as it was in Experiment 2 with the exception that the response-signal methodology was applied in the Simon task while the spatial compatibility/incompatibility tasks were “fast and accurate” standard RT tasks.

### Results and discussion

#### Simon task

***SAT analysis of task-relevant identity information.*** The *d*'_*id*_ vs. response lag data were fit using the standard SAT function (Equation 3) and the hyperbolic SAT function (Equation 4). Fit was quantitatively and qualitatively assessed using a hierarchical model-testing approach, commonly used in SAT studies (McElree and Dosher, 1989; Carrasco and McElree, 2001; Giordano et al., 2009). The models ranged from all factorial combinations that ranged from single fit (1 λ, 1 β, 1 δ, and 1 λ, 1 ω, 1 κ) to both datasets to a fully saturated model (2 λ, 2 β, 2 δ, and 2 λ, 2 ω, 2 κ). Model error was assessed using a least squares approach wherein normalized residuals were scaled to the total error for the model.

The analysis of the SAT data was accomplished in two stages. In the first stage, the best fit parameters of the group mean were identified for the compatibility and incompatibility groups separately. Goodness of fit was assessed with the adjusted *R*^2^ method (Dosher et al., 2004). These fit parameters were then used as starting points for the hierarchical modeling approach, where the mean data for both groups were concurrently fit using nonlinear data-fitting optimization routines (i.e., with the lsqnonlin function in Matlab; Mathworks Inc., Natick, MA). The second stage determined the best fit parameters for each individual participant using Equations 3 and 4. These parameter values were statistically compared across compatibility and incompatibility groups using unpaired *t*-tests.

**Figure 2:**
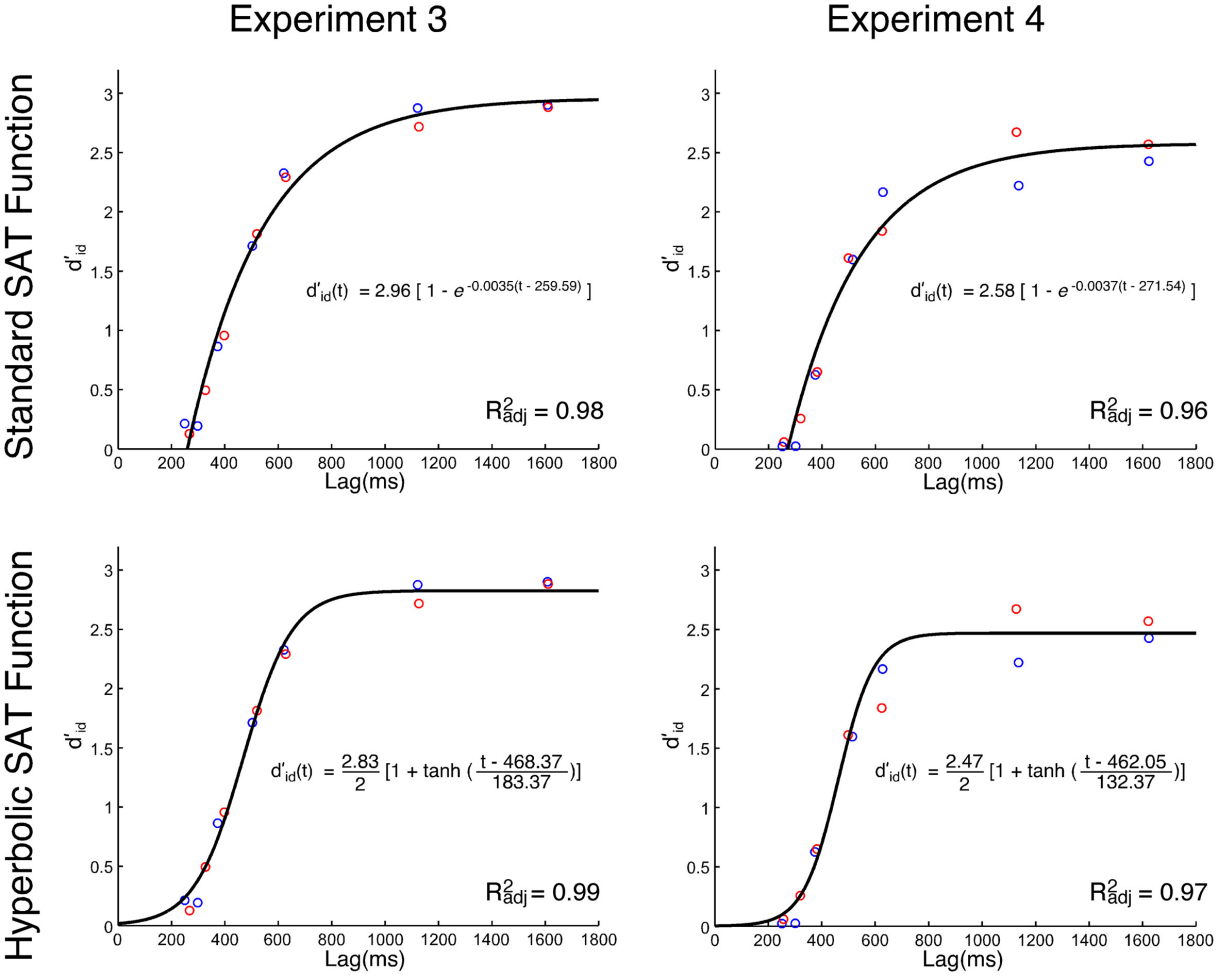
**Group mean *d*'_*id*_ values as a function of processing lag (ms) in Experiments 3 and 4. Top-row:** best fit of the standard speed-accuracy tradeoff function (Equation 3). **Bottom-row:** best fit of the hyperbolic tangent speed-accuracy tradeoff function (Equation 4). The Simon SAT data from the spatial incompatibility group are red. The Simon SAT data from the spatial compatibility group are blue.

The analysis of the *d*'_*id*_ data, using the standard SAT equation (Equation 3), revealed that the model with a single set of parameters (1 λ, 1 β, 1 δ) across the datasets for the spatial incompatibility and compatibility groups had the best fit overall (*R*^2^_*adj*_ = 0.98). The group mean, and the best fit, are presented in Figure 2. Equation 3 was then fit to the individual data for the compatible and incompatibility groups. In general, the fits were very good (average fit for compatibility group: *R*^2^_*adj*_ = 0.86; average fit for the incompatibility group: *R*^2^_*adj*_ = 0.86). The parameters from the fits for each group were compared and no differences were significant.

The hyperbolic equation (Equation 4) was also fit to the group mean. Again, a model that assumes a single set of parameters (1 λ, 1 ω, 1 κ) had the best fit (*R*^2^_*adj*_ = 0.99), slightly better than the fit of the standard SAT equation. The best hyperbolic fit and the group mean are presented in Figure 2. The individual fits for the spatial compatibility (mean *R*^2^_*adj*_ = 0.95) and incompatibility (mean *R*^2^_*adj*_ = 0.94) groups were also quite good. The parameters from the fits from each group were compared, and again, there were no significant differences.

**Figure 3:**
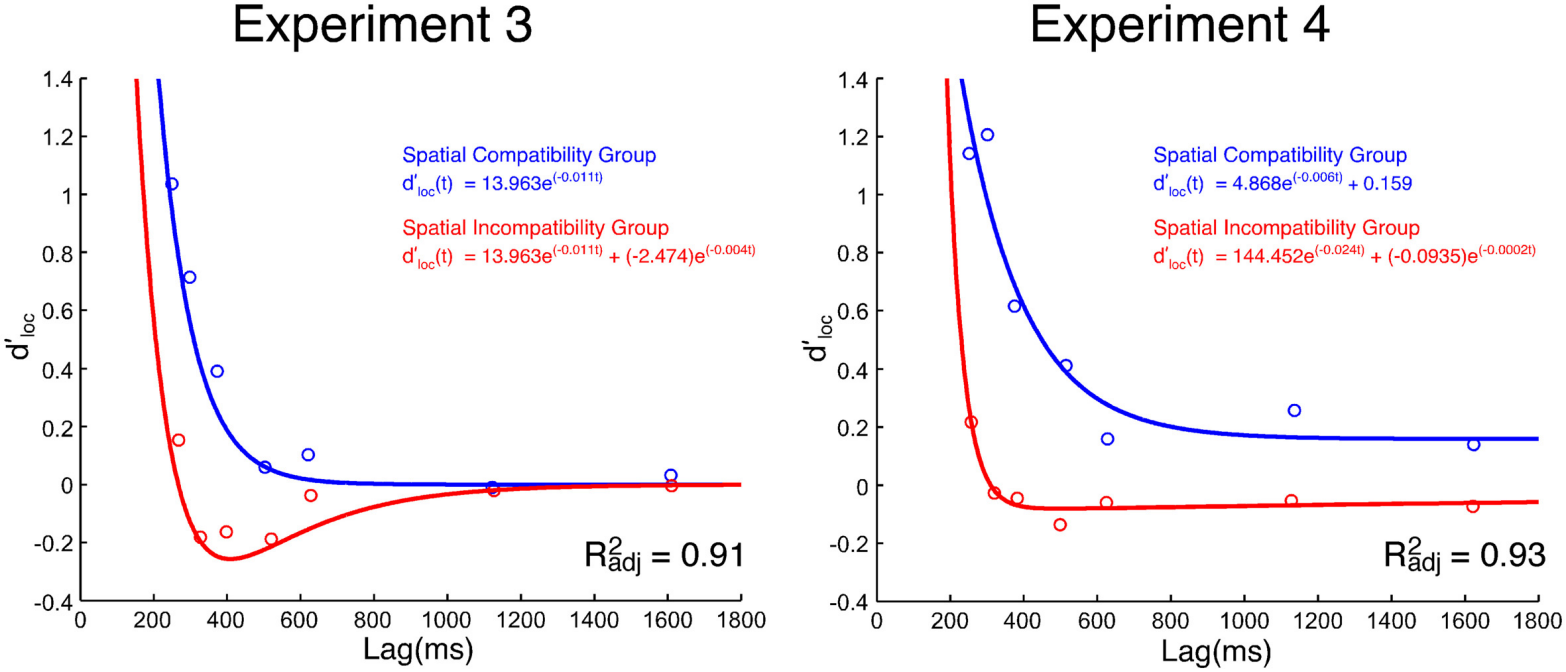
**Group mean *d*'_*loc*_ values as a function of processing lag (ms) in Experiments 3 and 4**. The Simon SAT data from the spatial incompatibility group are red. The Simon SAT data from the spatial compatibility group are blue.

***SAT analysis of task-irrelevant location information.*** Unlike the effect of task-relevant information (*d*'_*id*_) on response choice, the effect of location-based information (*d*'_*loc*_) lessened with time (see Figure 3). Neither the standard SAT (Equation 3) nor the hyperbolic (Equation 4) function fit the data particularly well. While Equation 5 (i.e., single exponential decay) fit the data for the compatibility group well, it failed to fit the data for the incompatibility group. As previously discussed, *d*'_*loc*_ reflects the impact of spatial information on response selection. At any given moment, *t, d*'_*loc*_ may be jointly influenced by the direct and/or indirect spatial pathways depicted in Figure 1. The activity along each spatial pathway is believed to lessen with time and have a summative effect on response selection. Thus, a second exponential component was included to account for these two sources of spatial information,

(7) $${d^{\prime }}_{loc}\left(t\right)=\delta_{1}e^{\left(\beta_{1}t\right)}+\;\delta_{2}e^{\left(\beta_{2}t\right)},\;for\;t>\;0.$$

Because the compatible and incompatible datasets could not be fit by the same function, we abandoned the hierarchical modeling approach. To analyze the decay of the task-irrelevant location information, we developed two models. The models were derived from the architecture depicted in Figure 1. Both models were fit to the mean group data using nonlinear data-fitting optimization routines in Matlab (Mathworks Inc., Natick, MA).

Both models presume that the *d*'_*loc*_ values in Simon tasks, when combined with an incompatibility task, are the result of two exponential functions (Equation 7): (1) a positive component resulting from the direct pathway, and (2) a negative component resulting from the spatial incompatible mapping (i.e., the indirect spatial pathway). The models only differ in their characterization of the S-R transfer from the spatial compatibility task to the Simon task.

The first model (Model 1) specifically presumes there is S-R transfer from the spatial compatibility task to the Simon task. The model holds that the component that is transferred from the spatial compatibility task to the Simon task is similar in magnitude, but opposite in direction (i.e., toward, not away, from the location), to the negative component passed along from the spatial incompatibility task to the Simon task (i.e., 1β_1_, 1δ_1_, 2β_2_, 1δ_2_; with the constraint that β_2_ in the spatial compatibility task is equal to -β_2_ in the spatial incompatibility task). This account presumes that there are not only two exponential components (Equation 7) in the spatial incompatibility group, but also two exponential components in the spatial compatibility group. The best fit for this model (Model 1) was quite poor (*R*^2^_*adj*_ = 0.38).

The second model (Model 2) presumes that, although the spatial incompatibility task introduces a third pathway to the Simon task, the spatial compatibility task has no effect on the Simon effect. There are only two studies (Proctor and Lu, 1999; Tagliabue et al., 2000) that have directly compared the Simon effect in a neutral condition to one that follows a spatial compatibility task. In both cases, there was no evidence of S-R transfer from a spatial compatibility task to the Simon task. Accordingly, Model 2 includes only a single exponential function for the Simon task *d*'_*loc*_ data in the spatial compatibility group (Equation 5: 1β_1_, 1δ_1_) and the same exponential component and a negative-going exponential component in the spatial incompatibility group (Equation 7: 1β_1_, 1δ_1_, β_2_, δ_2_). Thus, only one of the exponential components is shared, while the function for the incompatibility group also includes a second exponential component reflecting the third, indirect (residual) pathway. This model fit the group mean reasonably well (*R*^2^_*adj*_ = 0.91). The group mean is plotted in Figure 3 along with the fitted parameters from Model 2.

It was not possible to directly compare parameters from the group-derived models for the compatible and incompatible groups because the best fits were achieved with different functions. Moreover, the fits of Model 2 to the individual data were quite variable, with some being quite good (e.g., *R*^2^_*adj*_ = 0.97) and others failing to reach a meaningful convergence. Thus, as a second step in the analysis, we performed *post-hoc*, unpaired *t*-tests on the *d*'_*loc*_ values for each SOA. This approach does not presume any particular model. The *d*'_*loc*_ value was significantly greater for the compatibility group than the incompatibility group at the 60 ms [*t*_(28)_ = 3.33, *p* < 0.005], 120 ms [*t*_(28)_ = 3.68, *p* < 0.005], and 240 ms [*t*_(28)_ = 2.49, *p* < 0.05] SOAs. No other difference was significant. As shown in Table 2, none of the *d*'_*loc*_ values differed from 0 in the spatial incompatibility group while the *d*'_*loc*_ at the three earliest SOAs did differ from 0 in the spatial compatibility group.

The SAT analysis of the Simon task revealed two key findings. First, there were no effects of the spatial incompatibility task on *d*'_*id*_. The model fits and inferential statistics suggest that the spatial compatibility and incompatibility tasks had no impact on the ability to identify the task-relevant stimulus features (i.e., shape and/or orientation) in the Simon task. This finding is in accord with the model proposed in Figure 1 and suggests there is some independence between the indirect, residual pathway and the indirect, task-relevant pathway. Second, the spatial incompatibility task did have a noticeable effect on the sensitivity to the location of the stimulus (*d*'_*loc*_). The spatial incompatibility task appeared to weaken, but not reverse, the Simon effect (as measured with *d*'_*loc*_) in a SAT task. This pattern is similar to what was observed in Experiment 2 in a standard RT Simon task. Interestingly, the evidence for an early tendency to respond to the location of the stimulus, while clear in the spatial compatibility group, was mixed in the spatial incompatibility group. The inferential statistics suggest that the *d*'_*loc*_ data do not differ from zero^1^. The modeling work, however, suggests that an early exponential component is being masked by a second component. It is possible, that these effects are the result of reduced S-R transfer because of a mismatch between task contexts.

#### Spatial compatibility and incompatibility tasks

The RTs for the spatial compatibility and incompatibility tasks were analyzed with a 2 (group) × 2 (congruency) × 7 (SOA) ANOVA. The mean RTs of the spatial compatibility group (*M* = 292 ms) were significantly faster than those of the incompatibility group (*M* = 342 ms), *F*_(1, 28)_ = 8.46, *MSE* = 31539.06, *p* < 0.01. RTs also increased with increasing SOA, *F*_(6, 168)_ = 6.63, *MSE* = 1718.17, *p* < 0.0001. No other main effect or interaction was significant. Table 3 provides the non-significant mean congruency effects for the spatial compatibility and incompatibility tasks.

The *d*'_*loc*_ data were entered into a 2 (group) × 7 (SOA) ANOVA. As expected, there was a large group effect [*F*_(1, 28)_ = 2361.80, *MSE* = 0.93, *p* < 0.0001] indicating that participants were following directions (i.e., responding to the target's location in the spatial compatibility task and away from the target's location in the spatial incompatibility task). There was also a group X SOA interaction, *F*_(6, 168)_ = 5.47, *MSE* = 0.10, *p* < 0.0001. The SOA effect was only significant in the incompatibility group, *F*_(6, 84)_ = 5.18, *MSE* = 0.07, *p* < 0.0005. The same analysis was performed on the *d*'_*id*_ data. None of the effects were significant. None of the *d*'_*id*_ values differed significantly from zero (Table 4).

The key finding from the spatial compatibility and incompatibility tasks was the absence of a congruency effect on RTs and *d*'. The possibility that this was the result of the disparity in task context (SAT and standard RT) was addressed in Experiment 4 where both tasks were SAT tasks.

## Experiment 4

In this experiment the response-signal methodology was applied to both Simon and spatial compatibility/incompatibility tasks. Thus, like Experiment 1, the task contexts were identical.

### Methods

#### Participants

There were 15 undergraduate participants in the compatibility group and 15 in the incompatibility group. Participants earned course credit and small performance bonuses, as in the previous experiments.

#### Procedure

In this experiment, both the Simon and spatial compatibility tasks were subject to the response-signal methodology. Thus, the Simon task was identical to the Simon task in Experiment 3 and the spatial compatibility/incompatibility tasks were identical to the spatial compatibility/incompatibility tasks in Experiment 2. The same SOA was used in each block of Simon and spatial compatibility/incompatibility trials.

### Results and discussion

#### Simon task

***SAT analysis of task-relevant identity information.*** The analysis of the data using the standard SAT equation (Equation 3), revealed—again—that the single fit [1 λ, 1 β, 1 δ] model had the best fit (*R*^2^_*adj*_ = 0.96; see Figure 2). The model was fit to the individual data for the compatible and incompatibility groups. In general the fits were very good (average fit for compatibility group: *R*^2^_*adj*_ = 0.89; average fit for the incompatibility group: *R*^2^_*adj*_ = 0.87). The parameters from the fits for each group were compared and no differences were significant.

The *d*'_*id*_ analysis using the hyperbolic equation (Equation 4) was fit to the group using the hierarchical model-testing approach, as described above. Again, a model that assumes a single set of parameters (1 λ, 1 ω, 1 κ) had the best fit (*R*^2^_*adj*_ = 0.97), slightly better than the fit of the standard SAT equation. The average of the fits to individual data was also good for the compatible (mean *R*^2^_*adj*_ = 0.90) and incompatibility groups (mean *R*^2^_*adj*_ = 0.91). The only comparison between individual fits that was significant was that between the asymptote, λ, [*t*_(28)_ = 2.20, *p* < 0.05]. The mean asymptote of individual fits was slightly higher for the spatial incompatibility group (*M* = 2.66) than it was for the spatial compatibility group (*M* = 2.31). This difference is apparent in the mean *d*_*id*_ values presented in the late SOAs in Figure 2. A *post-hoc* analysis of the group differences in *d*'_*id*_ for each SOA only revealed a difference at the 120 ms [*t*_(28)_ = 2.21, *p* < 0.05] and the 960 ms [*t*_(28)_ = 2.16, *p* < 0.05] SOA, although these effects do not survive a Bonferroni correction for multiple comparisons. Thus, the evidence that the spatial compatibility task had an effect on the sensitivity to the non-spatial, task-relevant feature (*d*'_*id*_) of the target in the Simon task was generally poor, and mixed, at best.

***SAT analysis of task-irrelevant location information.*** The SAT functions of the group mean *d*'_*loc*_ values are presented in Figure 3. The first pass of fitting the group mean, using the same models in Experiment 3, was unsuccessful. Model 1 fit the data very poorly (*R*^2^_*adj*_ = 0.15). Model 2 fared better, but the fit was less than spectacular (*R*^2^_*adj*_ = 0.71). That the mean data for the spatial compatibility group did not return to the zero baseline likely explains these poor fits. Using Equation 5, but including a constant, for the spatial compatibility group did not improve Model 2 (*R*^2^_*adj*_ = 0.64). In fact, the best model was one where the group mean for the spatial compatibility group was fit with a constant and the group mean for the spatial incompatibility group was fit with Equation 7 independently (*R*^2^_*adj*_ = 0.93).

The *d*'_*loc*_ values were significantly different between the spatial compatibility and incompatibility groups at each SOA (*p*s < 0.05), with the exception of the 480 ms SOA. The *d*'_*loc*_ values were also compared to 0 for each group and SOA. The *d*'_*loc*_ values for the spatial compatibility group were significantly greater than 0 at all SOAs (*p*s < 0.05, uncorrected). For the spatial incompatibility group, the *d*'_*loc*_ value at the 60 ms SOA was significantly greater than 0 (*p* < 0.05, uncorrected) and at the 1440 ms SOA the *d*'_*loc*_ value was significantly less than 0 (*p* < 0.05, uncorrected; see Table 2)^2^.

The present findings suggest that there are fundamental differences between the temporal dynamics of task-irrelevant spatial information processing in Simon tasks when mixed with spatial compatibility and incompatibility tasks. Unlike Experiment 3, the model with the best fit was one in which there were no shared parameters between spatial compatibility and incompatibility groups in the Simon task. A potential implication of this fully saturated model is that the direct spatial pathway may be compromised by the spatial compatibility/incompatibility task.

#### Spatial compatibility and incompatibility tasks

Accuracy was near ceiling in all conditions, as it was in Experiment 2, so the data were not subjected to a curve-fitting procedure. The *d*'_*loc*_ values were entered into a 2 (group) × 7 (SOA) ANOVA (see Table 4 for means). The main effects of group [*F*_(1, 28)_ = 1524.45, *MSE* = 0.76, *p* < 0.0001], SOA [*F*_(6, 168)_ = 9.70, *MSE* = 0.32, *p* < 0.0001], and the interaction [*F*_(6, 168)_ = 8.70, *MSE* = 0.32, *p* < 0.0001] were all significant. The interaction was the result of a much larger SOA effect in the spatial incompatibility task than the spatial compatibility task.

The *d*'_*id*_ values were also entered into a 2 (group) × 7 (SOA) ANOVA. Only the SOA effect was significant, *F*_(6, 168)_ = 3.43, *MSE* = 0.032, *p* < 0.005. *d*'_*id*_ values increased with SOA. However, only the *d*'_*id*_ values at the 360 and 1440 ms SOA were significantly different from 0 (see Table 4).

## General discussion

When a spatial incompatibility task is intermixed with a Simon task, the Simon effect is reversed (Marble and Proctor, 2000; Proctor et al., 2000, 2003; Proctor and Vu, 2002). In Experiment 1, this finding was replicated in a different paradigm where tasks predictably alternated between spatial incompatibility and Simon tasks. The most common explanation for this finding is that the spatial incompatibility task activates an additional, indirect pathway that connects nodes representing spatial features of the stimulus with response nodes (Figure 1). The current work addressed three features of this paradigm: bidirectional S-R transfer across Simon and spatial compatibility/incompatibility tasks, the modulating effects of task context similarity on S-R transfer, and the time course of task-irrelevant S-R location information on response selection.

### Bidirectional S-R transfer between simon and spatial compatibility tasks

This was the first study to explore bidirectional S-R transfer between Simon and spatial compatibility/incompatibility tasks in the mixed-tasks paradigm. Evidence for S-R transfer from the spatial compatibility/incompatibility task to the Simon effect was evident in all experiments. In general, performing the spatial incompatibility task with the Simon task reduced or reversed the tendency to respond to the location of the stimulus. This pattern has been observed in a number of studies in a variety of different paradigms (e.g., Tagliabue et al., 2000, 2002; Marble and Proctor, 2000; Proctor et al., 2007, 2013; Proctor and Vu, 2009; Yamaguchi and Proctor, 2009).

The evidence for S-R transfer from the Simon task to the spatial compatibility/incompatibility task was best when task contexts (SAT or standard RT) matched. Congruent responses, in the spatial compatibility and incompatibility tasks, were those in which the response associated with the non-spatial identity of the stimulus in the Simon task matched the location of the stimulus. In Experiment 1, the congruency effect for the spatial compatibility group was a speed-accuracy tradeoff: responses were faster and less accurate for incongruent trials. On the other hand, for those participants in the spatial incompatibility condition, congruent trials were faster and more accurate than incongruent trials. In Experiment 3, when the task contexts did not match, there was no effect of congruency on RTs or *d*'_*id*_. Congruency effects were rarely seen in the *d*'_*id*_ measure in SAT tasks with response-signal methodology (Experiments 2 and 4). Thus, transfer from the Simon task to the spatial compatibility/incompatibility tasks was weak and sporadic, suggesting that S-R transfer between Simon and spatial compatibility/incompatibility is bidirectional and asymmetric. S-R transfer from spatial incompatibility tasks to the Simon task was much more convincing than S-R transfer in the other direction. It may be that the precedence for location information (Hillyard and Munte, 1984) offers greater opportunities to influence tasks wherein the task-relevant information comes from slower (non-spatial) S-R pathways. Further research is needed to assess the precise reason for asymmetrical S-R transfer. The clearest evidence, in the current work, for a congruency effect came when (i) responding was slow (i.e., with the spatial incompatibility task in Experiment 1 and with long SOAs in Experiment 4) and (ii) the two tasks shared a task context (i.e., in Experiments 1 and 4). The context of the task, thus, appears to play a key role in S-R transfer.

### Task-context dependent S-R transfer

Environmental context plays a critical role in memory performance. When features of the encoding environment match features of the retrieval environment, memory performance is generally better than when the environmental features do not match (Godden and Baddeley, 1975). Smith and Vela (2001) noted that manipulations that draw attention to the task or away from the environmental context tend to reduce task-dependent memory effects. Thus, context plays an important role when it is attended during encoding and retrieval.

Yamaguchi and Proctor (2009) observed evidence for context-dependent S-R transfer from a spatial incompatibility task to a Simon task when the response mode (key-press vs. joystick response) was the same for both tasks. Response modality (as in Yamaguchi and Proctor, 2009) is one feature of task context; yet the context of the task may also include other features. In the current work, response-signal (i.e., SAT) methodology affected S-R transfer in a context-dependent manner. The SAT task not only included the same stimuli presented in the standard RT task, but also included other task-relevant stimuli such as an auditory response-signal tone and post-response feedback. These features likely contributed to the unique context of the task and were quite different from the context of the standard RT task. The results of the present investigation support this claim. The spatial incompatibility task reversed the Simon effect in Experiment 1 (where both tasks were standard RT tasks), but not in Experiment 2 where the Simon (standard RT methodology) and spatial incompatibility (response-signal methodology) tasks were different. In the response-signal (SAT) Simon tasks (i.e., Experiments 3 and 4), there was evidence for a late reversal of *d*'_*loc*_ in Experiment 4 (task contexts match), but not in Experiment 3 (task contexts do not match). Together, the evidence suggests that the opportunity for S-R transfer is greatest when task features are closely matched. Moreover, the current works also demonstrates that the context of the task plays an important role in the mixed-task experimental design.

### The time course of task-irrelevant location information on response selection

A number of previous studies have used vincentizing approaches to study the time course of the Simon effect. The challenge with this approach is that it relies on differences in the shape of RT distributions (Zhang and Kornblum, 1997; Pratte et al., 2010; Schwarz and Miller, 2012). The shape of an RT distribution can be affected by a number of factors like fast guesses, fatigue, or inattention. It can be troubling if these factors differ systematically across conditions. It is, perhaps, even more troubling that distributional approaches, like vincentizing, completely ignore error rates. Wickelgren (1977) argued that it “… may not be defensible … to attempt to test quantitative theories of information processing dynamics … by functions which use reaction time as the sole dependent variable, without simultaneously predicting accuracy.” (p. 81). Thus, researchers should be cautious not to overvalue the contribution of vincentized approaches (e.g., delta plots) to the temporal dynamics of information processing.

The response-signal (SAT) approach is similar to another approach that has been commonly used to study the time course of the Simon effect (Ridderinkhof, 2002). Conditional accuracy functions (CAFs) partition RTs, and error rates, into a small number of bins (Wood and Jennings, 1976; Ridderinkhof, 2002; Band et al., 2003), not unlike the vincentization approach. This analytic approach produces the so-called *micro*-SAT (Pachella, 1974). Micro-SAT analyses have also depicted the influence of task-irrelevant spatial information on response selection as an exponential decay function (e.g., Ridderinkhof, 2002). This approach, while less cumbersome than a full SAT analysis, may be criticized on two grounds. First, it can be argued that, not unlike vincentizing, different processes (guesses, fatigue, inattention, etc.) are not equally represented along the RT distribution. The response-signal approach avoids this pitfall by capturing a point along the SAT within a single block of trials. Secondly, as Pachella (1974) pointed out, the relationship between a micro-SAT and the standard SAT (sometimes called the *macro*-SAT) is unknown, but what is known is that they do not seem to tap into the same underlying function (Luce, 1986). Given this, some caution when interpreting CAFs is warranted (Wickelgren, 1975, 1977).

The current work extended the first SAT analysis of the Simon effect presented by Hilchey et al. (2011). A dissociation between two measures of sensitivity, *d*'_*id*_ (sensitivity to the task-relevant target feature) and *d*'_*loc*_ (sensitivity to the task-irrelevant spatial feature of the target), in the context of a Simon task was revealled. While *d*'_*id*_ increased with time (a standard SAT), *d*'_*loc*_ decreased with time. The *d*'_*id*_ data were fit with the standard SAT function and a hyperbolic tangent function. Both fits were excellent, although the hyperbolic tangent function fit was slightly superior. This is not to suggest that the hyperbolic tangent function should replace the standard SAT function. Future research is needed to determine which function might best describe performance in a wider range of tasks.

The spatial incompatibility task had virtually no impact on *d*'_*id*_ in the Simon task, suggesting independence between spatial S-R transfer and the processes involved in the identification of non-spatial, task-relevant, target features. The only fly in the ointment was seen in the hyperbolic tangent fits in Experiment 4: the asymptotic parameter (λ) was significantly higher for the spatial incompatibility group than the spatial compatibility group. There are, however, a number of reasons to be skeptical about this finding. First, there was little reason to expect, from any *a priori* theoretical perspective, that the ability to identify the non-spatial, task-relevant feature in a Simon task should be better when the alternate task is a spatial incompatibility task than a spatial compatibility task. Second, there was no significant difference between the asymptotic parameters, derived from the standard SAT function (Equation 3), for the spatial compatibility and incompatibility groups in both Experiments 3 and 4. Third, a *post-hoc* analysis suggested the *d*'_*id*_ difference between groups was only significant at one of the late SOAs (960 ms) near asymptote. Lastly, in Experiments 3 and 4 the best fits to the *d*'_*id*_ group data in the Simon task assumed only a single set of parameters, suggesting the alternate task (i.e., the spatial compatibility or incompatibility task) had no impact on the accumulation of task-relevant information. Thus, the asymptotic difference between the groups found in Experiment 4 is, at best, equivocal.

Although the spatial incompatibility task had no influence on *d*'_*id*_ in the Simon task, it had a robust effect on *d*'_*loc*_. This effect provides another example of a single dissociation between *d*'_*loc*_ and *d*'_*id*_. The *d*'_*loc*_ data were fit with an exponential decay function. The exponential models used in the current investigation were simply initial attempts at providing a quantitative description of the time course of task-irrelevant, spatial S-R activity. It could be argued that an exponential decay model is psychophysically implausible, as irrelevant S-R location information should follow a Gaussian, biphasic, accumulation-decay pattern (e.g., Kornblum et al., 1999). Unfortunately we did not capture an early accumulation phase. Future SAT investigations of the Simon effect may consider manipulations (e.g., Ivanoff et al., 2002) that might possibly delay the *d*'_*loc*_ function in order to capture an early accumulation phase. For now, it is worth noting that the decrease in *d*'_*loc*_ with time was fit reasonably well with an exponential decay model.

The pattern of *d*'_*loc*_ across time lag in the Simon task with the spatial compatibility group is very similar to the pattern of vincentized RTs for Simon effects when there is prior or concurrent experience with a spatial compatibility task (Tagliabue et al., 2000; Proctor and Vu, 2009; Proctor et al., 2013). Both approaches demonstrate a standard pattern of declining influence of task-irrelevant location information on response selection with time. In the current work, that a single exponential component described the time course of *d*'_*loc*_ in the Simon task with the spatial compatibility group is consistent with at least three mechanisms. First, there may be no S-R transfer from spatial compatibility tasks to Simon tasks. This proposal is consistent with Tagliabue et al's (2000) assertion that spatial compatibility tasks have no impact on the direct spatial pathway. Second, a spatial compatibility task may induce some activity along an indirect spatial pathway that is largely masked by robust activity along the spatial direct pathway. It is possible that this activity may be unmasked at later SOAs given conditions that favor S-R transfer. The evidence for this possibility comes from Experiment 4, where the best fit to the *d*'_*loc*_ Simon data for the spatial compatibility group included a constant because *d*'_*loc*_ did not decline to zero. Unfortunately, this particular finding is ambiguous and may be explained by another mechanism. It is possible that the spatial compatibility task modulates the decline (decay or suppression) of the spatial direct pathway. This account is generally consistent with Proctor and Lu's (1999) original proposal that the direct spatial pathway is not “unmodifiable.” It is not consistent, however, with some modeling approaches (e.g., Tagliabue et al., 2000). Future research is needed to disentangle and dissociate the effects of different spatial S-R pathways on response decisions.

Perhaps the most important contribution of the current work stemmed from the observation that the *d*'_*loc*_ time course in the Simon task was different across the spatial compatibility/incompatibility groups. The time course of *d*'_*loc*_ in the Simon task, performed concomitantly with a spatial incompatibility task, was unlike that observed with previous research using vincentization approaches (Marble and Proctor, 2000; Tagliabue et al., 2000; Proctor and Vu, 2009) where the reverse Simon effect generally increased with time. There was no evidence for a monotonically increasing reverse Simon effect in the current study. In Experiment 3, although none of the *d*'_*loc*_ values in the Simon task differed significantly from zero across SOAs, the spatial incompatibility group Simon data were fit well to a model that included two exponential decay components: (i) the identical exponential decay component found in the spatial compatibility group, presumably capturing activity along the spatial direct pathway, and (ii) a negative exponential decay component that captured a slight tendency to respond away from the location of the stimulus. In Experiment 4, the spatial incompatibility group mean data were also fit to a double exponential function (Equation 7) quite well. Moreover, in Experiment 4, the *d*'_*loc*_ value of the spatial incompatibility group at the earliest SOA was greater than 0, indicating a tendency to respond to the location of the stimulus). Interestingly, at the longest SOA, the opposite pattern emerged (i.e., indicating a tendency to respond away from the location of the stimulus). The data-fitting approaches espoused herein appeared to be particularly sensitive to the time course of *d*'_*loc*_ and the findings generally support the tripartite pathway model depicted in Figure 1. In summary, the findings from the current study suggest that the early activity along the direct, task-irrelevant, spatial S-R pathway is indeed masked by late (and relatively persistent) residual activity from the indirect spatial S-R pathway. The current findings are also consistent with modeling approaches that presume activity along the task-irrelevant direct spatial pathway is unaffected by prior, or concurrent, experience with a spatial incompatibility task (Tagliabue et al., 2000).

## Conclusions

The present findings firmly establish Simon's (1969) original claim that there is a natural tendency to respond toward the source of stimulation. Performing a spatial incompatibility task can reverse or eliminate this tendency. However, the current results suggest that activity along the indirect spatial pathway may mask this natural tendency to respond to the source of stimulation. The present work also suggests that response-signal (i.e., SAT) methodology provides a task context that that may promote or impede S-R transfer. Lastly, these findings also demonstrate that transfer between spatial compatibility/incompatibility tasks and the Simon task can be bidirectional, although asymmetric.

### Conflict of interest statement

The authors declare that the research was conducted in the absence of any commercial or financial relationships that could be construed as a potential conflict of interest.

## Footnotes

^1^Given that there was no significant impact of task-irrelevant spatial information on responding for the spatial incompatibility group, fit was assessed with a third model (Model 3). This model assumes a single exponential function (Equation 5) for the spatial compatibility group and no impact (i.e., *d*'_*loc*_ = 0) of task-irrelevant spatial responding for the spatial incompatibility group. Model 3 yielded a reasonable fit (*R*^2^_*adj*_ = 0.91), virtually indistinguishable from Model 2. However, when the spatial compatibility group was excluded from the analysis, the nil model (*d*'_*loc*_ = 0) fit the data of the spatial incompatibility group more poorly (*R*^2^_*adj*_ = −0.11) than a double exponential model (Equation 7; *R*^2^_*adj*_ = 0.85), suggesting that any description of the data presuming there is no effect of task-irrelevant spatial information on response decisions is likely false.

^2^When the mean for the spatial incompatibility group was fit to a nil model (i.e., *d*'_*loc*_ = 0), as in Experiment 3 (see Footnote 1), and the spatial compatibility group mean was fit with a single exponential function (Equation 5; with a constant), it produced a reasonable fit (*R*^2^_*adj*_ = 0.92). However, excluding the spatial compatibility group from the analysis, again, tells a different story. The fit of the spatial incompatibility group mean to a nil model was poor (*R*^2^_*adj*_ = 0.09), while the fit to a double exponential function (Equation 7) was good (*R*^2^_*adj*_ = 0.88). Once more, any description of the data suggesting there is no effect of task-irrelevant spatial information on responding is likely false.
